# Combined Effects of Amikacin and Methylene Blue-Mediated Photodynamic Therapy on *Pseudomonas aeruginosa* Biofilms Mimicking Mono- and Polymicrobial Diabetic Foot Ulcer Infections

**DOI:** 10.3390/pathogens15020226

**Published:** 2026-02-18

**Authors:** Florencia Mariani, Celeste R. Costilla, Oscar J. Oppezzo, Estela M. Galvan

**Affiliations:** 1 Laboratorio de Patogénesis Bacteriana, Departamento de Investigaciones Bioquímicas y Farmacéuticas, Centro de Estudios Biomédicos, Biotecnológicos, Ambientales y Diagnóstico (CEBBAD), Universidad Maimónides, Hidalgo 775, Buenos Aires C1405BCK, Argentinacostilla.celeste@maimonides.edu (C.R.C.); 2 Consejo Nacional de Investigaciones Científicas y Técnicas (CONICET), Buenos Aires C1425FQB, Argentina; 3 Comisión Nacional de Energía Atómica, Buenos Aires C1429BNP, Argentina

**Keywords:** diabetic foot ulcer, biofilm, *Pseudomonas aeruginosa*, sequential antibiofilm treatment, antibiotics, methylene blue-mediated photodynamic therapy

## Abstract

Diabetic foot ulcer (DFU) infections frequently involve biofilm formation and exhibit limited responsiveness to conventional antibiotic therapy. In particular, *Pseudomonas aeruginosa* often participates in mono- and polymicrobial biofilms that display high tolerance to antimicrobial agents. This study evaluated the efficacy of methylene blue-mediated antimicrobial photodynamic therapy (aPDT), alone and in combination with antibiotics, against *P. aeruginosa* biofilms formed either as single-species or in mixed communities with *Enterococcus faecalis*, under conditions mimicking DFU infections. Macrocolony biofilms were challenged with amikacin alone (for single-species biofilms) or amikacin plus ampicillin (for mixed biofilms), aPDT, or sequential combinations of these treatments, and bacterial viability was quantified by colony-forming unit enumeration. Antibiotic treatment alone produced only modest reductions in *P. aeruginosa* viability, even at high concentrations, while aPDT using methylene blue was effective only at high photosensitizer concentrations. In contrast, sequential treatment with antibiotics followed by aPDT and a second antibiotic challenge resulted in a marked reduction in *P. aeruginosa* viability in both mono- and polymicrobial biofilms. Scanning electron microscopy revealed extensive structural damage in *P. aeruginosa* cells following combined treatments, whereas *E. faecalis* remained unaffected. Overall, our findings demonstrate that combining aPDT with antibiotics significantly enhances antibiofilm activity against *P. aeruginosa*, highlighting this approach as a promising alternative for the management of biofilm-associated DFU infections.

## 1. Introduction

Diabetic foot ulcers (DFUs) represent a serious and prevalent complication of diabetes, with an estimated lifetime incidence of 19–34% among affected individuals. This condition is associated with substantial morbidity, increased mortality risk, and considerable healthcare expenditures on a global scale [1]. The pathogenesis of DFU involves a multifactorial interplay among neuropathy, peripheral vascular disease, and impaired wound healing, all of which are further exacerbated by hyperglycemia. This combination creates a favorable environment for microbial colonization and subsequent infection, with approximately 50% of DFUs progressing to an infection state [2].

Patients with chronically infected DFUs, extensive necrosis, wet gangrene, and prolonged use of antibiotics have mixed microbial etiologies involving Gram-positive cocci, Gram-negative bacteria, and anaerobes [3]. Moreover, the microbial composition of DFU infections has been reported to vary across geographical regions [4]. In this context, a high prevalence of *Enterococcus faecalis* was observed in DFU patients from an Argentine hospital, frequently co-isolated with *Escherichia coli*, *Morganella morganii*, and *Pseudomonas aeruginosa* [5].

Polymicrobial biofilms arise in chronically infected DFUs, supported by a microenvironment that offers optimal conditions for their establishment and persistence [6,7]. Bacterial biofilms are microbial communities of cells attached to biotic or abiotic surfaces, and embedded in a self-produced extracellular polymeric matrix [8]. It has been reported that diabetic patients experience reduced peripheral blood supply, which further hinders the ability of the immune system and antibiotic treatments to eradicate these biofilms [9]. This aligns with evidence demonstrating the limited efficacy of antibiotic therapy in infections involving biofilms [10]. This challenge is further intensified in polymicrobial biofilms, where synergistic interspecies interactions have been reported to confer increased resistance to antimicrobial treatments [11]. In particular, tolerance and resistance of *P. aeruginosa* biofilms to several antibiotics have been reported, raising the need for alternative therapeutic approaches [12]. Nevertheless, whether interactions between *P. aeruginosa* and *E. faecalis*, a clinically relevant partner species in DFU infections, influence antibiotic susceptibility has not been fully explored.

Antimicrobial photodynamic therapy (aPDT) has been proposed as a therapeutic strategy against wound infections [13]. In photodynamic therapy, a light source of a specific wavelength is used to irradiate non-toxic photosensitizers such as tetrapyrroles, synthetic dyes, or naturally occurring compounds to generate reactive oxygen species that can exert a lethal effect on the microbe. Among available photosensitizing compounds, methylene blue (MB) is extensively utilized due to its low cytotoxicity and favorable safety profile in photodynamic therapy [14]. Topical or localized delivery of photosensitizers results in their rapid interaction with both Gram-positive and Gram-negative microorganisms and has demonstrated substantial efficacy in controlling infections and promoting accelerated wound healing [15]. Moreover, aPDT has shown great potential against biofilms, both as an independent treatment and when used in combination with antibiotics [16].

Although aPDT has shown promising activity against some biofilm models, its effects either as a single treatment or in combination with antibiotics on biofilms associated with DFU infections remain insufficiently characterized. In particular, it is not yet clear whether MB–mediated aPDT can enhance antibiotic activity against *Pseudomonas aeruginosa* in single- and mixed-species biofilms involving *Enterococcus faecalis* grown under DFU-like conditions. Accordingly, we hypothesized that MB-mediated aPDT may potentiate the antibacterial effect of conventional antibiotics, leading to improved killing of *P. aeruginosa* in both single- and mixed-species biofilms. To test this hypothesis, the efficacy of aPDT alone and in sequential combination with antibiotics (amikacin alone for *P. aeruginosa* single-species biofilms or amikacin plus ampicillin for mixed biofilms) was evaluated against macrocolony biofilms formed under conditions mimicking DFU infections.

## 2. Materials and Methods

### 2.1. Bacterial Strains and Inoculum Preparation

*P. aeruginosa* Pa036 and *E. faecalis* Ef037 were co-isolated from a patient with polymicrobial DFU at Hospital de Clínicas, Buenos Aires, Argentina [5]. Pa036 was susceptible to amikacin, piperacillin-tazobactam, ceftazidime, imipenem, meropenem, and colistin, and resistant to cefepime, ciprofloxacin, levofloxacin, and gentamicin. Meanwhile, Ef037 was susceptible to ampicillin, ampicillin-sulbactam, vancomycin, teicoplanin, ciprofloxacin, linezolid, and tigecycline, and resistant to minocycline. No prior antibiotic administration to the patient was reported before the isolation of the two pathogens. The isolates were maintained in the laboratory as frozen stocks (at −80 °C) in Tryptic soy broth (TSB) (Britania, Buenos Aires, Argentina) supplemented with 15% glycerol.

Inocula for both biofilm formation assays and planktonic growth were prepared as follows. For each experiment, strains were freshly streaked in TSB-agar (TSA) plates and grown overnight at 37 °C. Subsequently, individual colonies from each strain were used to inoculate TSB medium, and cells were incubated overnight at 37 °C and 200 rpm. Bacterial concentration (colony-forming units (CFUs)/mL) were determined from optical density measurements at 600 nm (OD_600_) as described previously [5]. Then, for single-species inocula, each culture was diluted in Lubbock medium (TSB with 50% bovine plasma and 5% freeze–thaw laked horse red blood cells) [17] supplemented with 1% glucose (Glc) in order to obtain a final concentration of 1 × 10^8^ cells/mL (for macrocolony biofilm assays) and 1 × 10^6^ cells/mL (for planktonic assays). For mixed inocula, equal volumes (1:1) of each single-species inoculum in Lubbock-Glc were combined.

### 2.2. Determination of Minimum Inhibitory and Bactericidal Concentrations in Planktonic Cultures of P. aeruginosa and E. faecalis

The activities of amikacin (Amk) and ampicillin (Amp) (Sigma-Aldrich, St. Louis, MO, USA) against planktonic cultures of *Pseudomonas aeruginosa* Pa036 and *Enterococcus faecalis* Ef037 were assessed. Minimum inhibitory concentrations (MICs) and minimum bactericidal concentrations (MBCs) were determined by the broth microdilution method in Mueller–Hinton (MH) broth, following the guidelines of the Clinical and Laboratory Standards Institute [18]. Assays were conducted in sterile 96-well microplates with a final volume of 200 μL per well. Bacterial suspensions were adjusted to 1 × 10^6^ CFU/mL and inoculated into MH medium containing increasing concentrations of the antibiotic under evaluation. Plates were incubated at 37 °C for 24 h under agitation (200 rpm). Bacterial growth was quantified by measuring the optical density at 600 nm (OD_600_) using a μQuant™ microplate spectrophotometer (BioTek, Winooski, VT, USA). The MIC was defined as the lowest antimicrobial concentration resulting in 95% ± 5% inhibition of bacterial growth. To determine the MBC, tenfold serial dilutions were prepared from wells exhibiting no visible turbidity, followed by colony-forming unit (CFU) enumeration as described in Section 2.5. The MBC was defined as the lowest antimicrobial concentration yielding a ≥99.9% (3 Log_10_ CFU) reduction in viable cell counts.

### 2.3. Macrocolony Biofilm Assays

Macrocolony biofilms were formed as previously described [5], with minor modifications. Briefly, 5 µL of the corresponding bacterial inoculum (single- and mixed-species) containing 5 × 10^5^ cells was spotted on sterile membrane discs (1 cm diameter, 0.2 µm porosity, mixed cellulose ester membrane, Millipore, Burlington, MA, USA) placed onto Lubbock-Glc-1.7% agar plates and then incubated at 37 °C for 24 h. Formed single- and dual-species macrocolonies were subjected to antimicrobial treatments as explained below.

### 2.4. Biofilm Challenge Using Antibiotics, Antimicrobial Photodynamic Therapy (aPDT) and Combined Treatments

Macrocolonies grown on membrane discs, as explained above, were exposed to varying antibiotic concentrations by transferring the membranes onto MH-agar plates supplemented with the corresponding antibiotic. The plates were then incubated for an additional 24 h at 37 °C. Macrocolony biofilms placed on MH-agar plates without antibiotics served as control. Single-species Pa036 biofilms were challenged with Amk (2.5–250 µg/mL). Dual-species Pa036-Ef037 macrocolonies were exposed to Amk (2.5–250 µg/mL), Amp (3.0–3000 µg/mL), or a mixture of both antibiotics (Amk 25 µg/mL + Amp 75 µg/mL). When indicated, an additional 24 h challenge with the same antibiotic condition was performed.

For aPDT, a 6 mM methylene blue (MB) stock solution was prepared in PBS, filter-sterilized and stored at room temperature in the dark. Tested MB concentrations were 60 µM, 600 µM, and 6 mM. For each assay, 20 µL of MB were applied onto the macrocolonies and incubated in the dark for 2 h at room temperature. Then, the macrocolonies were placed on a 1.7% agar-agar plate and irradiated for 2 h with red LED light. For this, arrays of light-emitting diodes which emitted in a narrow band of about 637 nm were used as radiation sources [19]. The fluence rate at the free surface of the bacterial suspensions was 44 Wm^−2^.

For selected experiments, sequential treatments with antibiotics and aPDT were carried out.

After treatments, macrocolonies were mechanically disrupted in sterile 0.9% NaCl solution. For CFU enumeration was performed as described in Section 2.5.

### 2.5. Quantification of Cultivable Cells

The number of cultivable cells from disrupted biofilms and planktonic cultures was determined by CFU counts. For that purpose, bacteria were serially diluted (1:10) and plated on agar plates. Single-species samples were plated on TSB-agar. To distinguish between *P. aeruginosa* Pa036 and *E. faecalis* Ef037 in mixed samples, Bile Esculin Azide Agar (Britania, Buenos Aires, Argentina) (selective for *E. faecalis*) or TSB-agar plates supplemented with 30 μg/mL ampicillin (selective for *P. aeruginosa*) were used [5].

### 2.6. Scanning Electron Microscopy (SEM)

Macrocolony biofilms were processed for SEM as described by Serra et.al. [20]. Briefly, macrocolonies on membrane discs were placed in glass dishes containing 1% (wt/vol) osmium tetroxide in 0.05 M cacodylate buffer (pH 7.5), and incubated for 45 min at room temperature to allow fixation to proceed. After washing with a cacodylate buffer, macrocolonies were subjected to dehydration in a graded alcohol series (30, 50, 70, 90, and 100% ethanol). The dehydrated specimens were dried in a critical point drier (CPD Polaron, London, UK) with liquid carbon dioxide as a transitional fluid. After the drying, macrocolonies were mounted on aluminum stubs using double-sided adhesive tape and coated with gold in a sputter coater (Pelco 9100 Sputter Coater, Redding, CA, USA). The specimens were examined with a Zeiss GeminiSEM 360 (ZEISS, Jena, Germany) scanning electron microscope operating at an accelerating voltage of 5 kV under high vacuum mode. At least two macrocolonies derived from independent cultures were examined per condition.

### 2.7. Statistical Analysis

Statistical significance was determined by one-way ANOVA with Tukey’s tests for multiple comparison. A *p*-value < 0.05 was considered significant. Analyses were performed using GraphPad Prism, version 9 (GraphPad Software, San Diego, CA, USA).

## 3. Results

### 3.1. Evaluation of Single-Species P. aeruginosa Biofilm Susceptibility to Antimicrobial Treatments

#### 3.1.1. Effect of Amikacin on Preformed *P. aeruginosa* Biofilms

**Figure 1:**
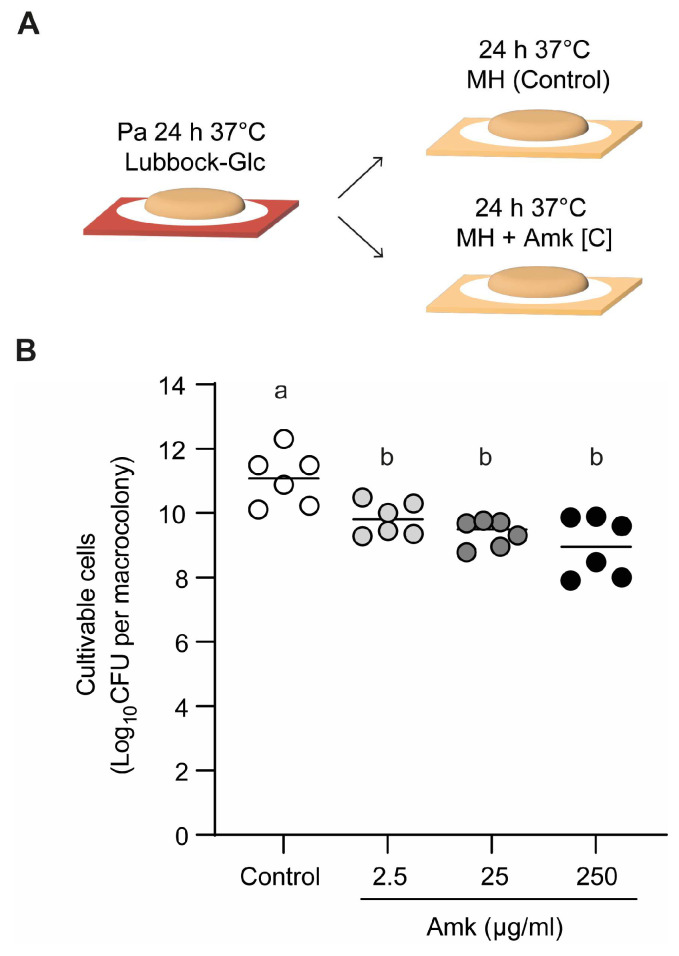
Effect of amikacin exposure on cell viability of *P. aeruginosa* macrocolony biofilms. (**A**) Schematic of macrocolony biofilm development on membranes placed on Lubbock-Glc agar during 24 h, followed by 24 h amikacin treatment on MH agar. Control corresponds to biofilms placed on MH agar without antibiotic. (**B**) Viable cell enumeration of biofilm-associated bacteria after exposure or not to increasing amikacin concentrations. In each case, N = 6 independent experiments. Different letters indicate *p* < 0.05 assessed by one-way ANOVA followed by Tukey’s post hoc test.

*P. aeruginosa*, a microorganism commonly associated with diabetic foot ulcer (DFU) infections and known to form robust biofilms, was chosen. In particular, the clinical isolate Pa036, which is sensitive to amikacin, was selected. The minimum inhibitory and bactericidal concentrations (MIC and MBC) to amikacin under planktonic growth conditions were first determined using the standard microdilution method in MH broth, yielding values of 0.78 µg/mL and 2.50 µg/mL, respectively. Considering that biofilm-associated bacteria are typically less susceptible to antibiotics than their planktonic counterparts [10], Pa036 biofilms were exposed to increasing concentrations of amikacin, starting from the planktonic MBC (2.50 µg/mL). To this end, Pa036 macrocolony biofilms were first established for 24 h at 37 °C on membranes placed on Lubbock-Glc-agar. The membranes were then transferred onto MH-agar plates, supplemented or not with the corresponding amikacin concentration, and incubated for an additional 24 h (Figure 1A). Control biofilms reached 11.1 ± 0.8 Log_10_ CFU, whereas amikacin exposure led to only a modest reduction in viability in single-species *P. aeruginosa* biofilms. CFU enumeration revealed decreases of approximately 1–2 Log_10_ CFU across all tested amikacin concentrations, even when the antibiotic was applied at 100-fold the planktonic MBC (Figure 1B).

#### 3.1.2. Methylene Blue-Mediated Antimicrobial Photodynamic Treatment of *P. aeruginosa* Biofilms

**Figure 2:**
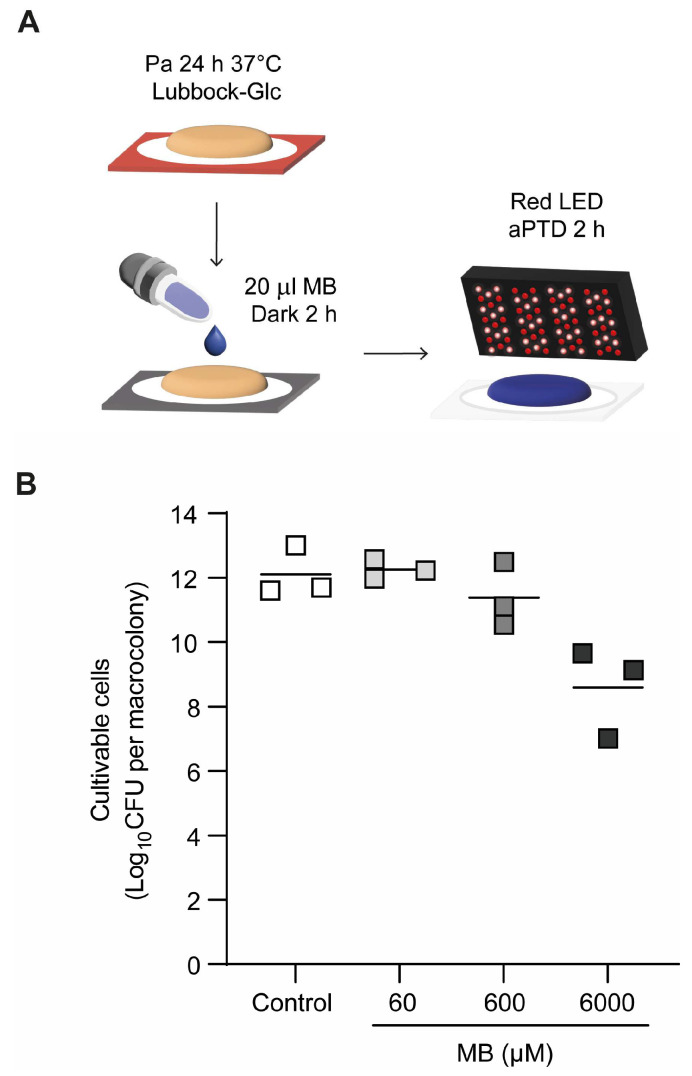
Effect of MB-mediated aPDT on cell viability of *P. aeruginosa* macrocolony biofilms. (**A**) Schematic of macrocolony biofilm development on membranes placed on Lubbock-Glc agar during 24 h, followed by methylene blue (MB) application, 2 h incubation in the dark, and 2 h irradiation with a red LED light source (λ_max_ 639 nm). (**B**) Viable cell enumeration of biofilm-associated bacteria after exposure or not to aPDT with increasing MB concentrations. In each case, N = 3 independent experiments. No significant differences were found among groups after one-way ANOVA analysis followed by Tukey’s post hoc test.

As an alternative to antibiotic treatment, antimicrobial photodynamic therapy (aPDT) was evaluated using methylene blue (MB) as the photosensitizer together with red LED irradiation. Pa036 biofilms were generated as explained above, after which the macrocolonies were exposed to increasing MB concentrations (from 60 to 6000 µM) by applying a 20 µL drop onto each macrocolony and incubating for 2 h in the dark to allow photosensitizer uptake prior to irradiation (Figure 2A). The macrocolonies were then irradiated for 2 h with a red LED light source (λ_max_ 639 nm), and post-treatment cell viability was quantified by CFU enumeration. Results showed that bacterial viability in untreated control Pa036 biofilms was comparable to that observed in Pa036 biofilms subjected to aPDT with MB concentrations up to 600 µM (Figure 2B). Among the tested conditions, only the highest MB concentration (6000 µM) produced a significant decrease in *P. aeruginosa* viability, yielding a reduction of 3.5 Log_10_ CFU relative to the controls (8.6 ± 1.4 vs. 12.1 ± 0.8).

#### 3.1.3. Assessment of *P. aeruginosa* Biofilm Susceptibility to Combined Amikacin and aPDT Treatments

**Figure 3:**
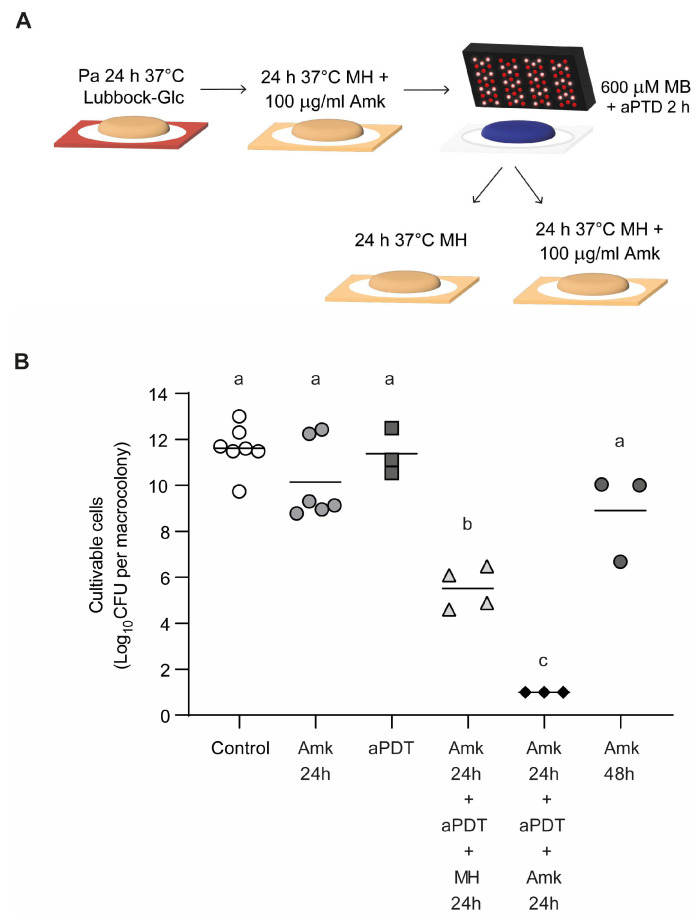
Effect of combined amikacin and MB-mediated aPDT on cell viability of *P. aeruginosa* macrocolony biofilms. (**A**) Schematic of sequential antimicrobial treatments of macrocolony biofilms developed on Lubbock-Glc agar (schematic of individual treatments were described earlier). Macrocolonies were sequentially exposed to: amikacin (100 μg/mL) for 24 h; aPDT with MB (600 μM) for 2 h; and either a second challenge with amikacin (100 μg/mL) or incubation in antibiotic-free MH agar. (**B**) Viable cell enumeration of biofilm-associated bacteria after the different antimicrobial treatments. In each case, N ≥ 3 independent experiments. Different letters indicate *p* < 0.05 assessed by one-way ANOVA followed by Tukey’s post hoc test.

We next assessed whether sequential application of amikacin treatment and MB-mediated aPDT enhanced the reduction of biofilm cell viability compared with either treatment alone. To this end, sublethal concentrations were selected: 100 µg/mL of amikacin and 600 µM MB for aPDT. As expected, individual treatments—either 24 h exposure to amikacin or 2 h of aPDT—did not significantly impact the viability of Pa036 single-species biofilms (Figure 3). In contrast, treatment with 100 μg/mL amikacin for 24 h followed by aPDT, and subsequently allowing the biofilms to recover for 24 h in antibiotic-free MH medium, resulted in a marked 6.1 Log_10_ CFU reduction relative to the untreated control (5.5 ± 0.9 vs. 11.6 ± 1.0 Log_10_ CFU). A greater effect was achieved when amikacin was applied, followed by aPDT and a second amikacin exposure, after which no viable cells were detected in the biofilm (counts below 1.0 Log_10_ CFU). Notably, treatment with amikacin alone for 48 h was less effective, producing only a 2.7 Log_10_ CFU decrease compared with the untreated control (8.9 ± 1.9 vs. 11.6 ± 1.0 Log_10_ CFU).

#### 3.1.4. Scanning Electron Microscopy Analysis of Morphological Alterations in *P. aeruginosa* Biofilms Following Antimicrobial Treatments

**Figure 4:**
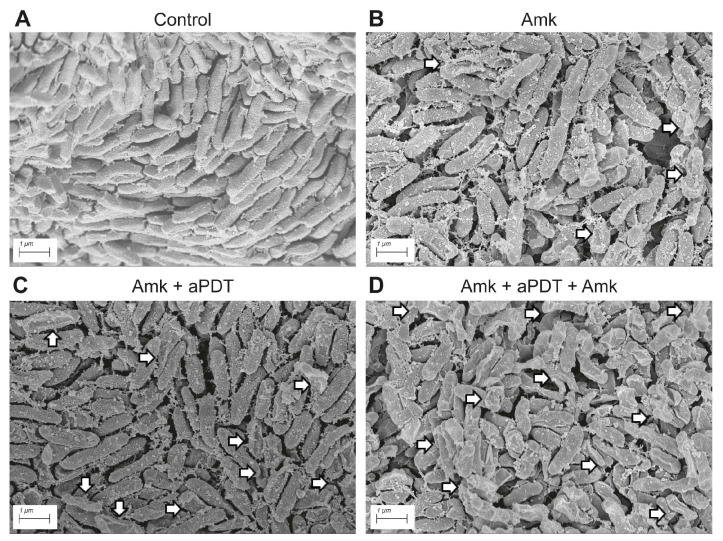
Scanning electron micrograph of *P. aeruginosa* macrocolony biofilms subjected to different antimicrobial treatments. Each micrograph corresponds to 24h-Pa036 biofilms under conditions explained in legend of Figure 3. (**A**) Untreated control; (**B**) Exposure to 100 μg/mL amikacin (Amk); (**C**) Sequential challenge with 100 μg/mL Amk followed by aPDT using 600 µM methylene blue (MB); (**D**) Same conditions as (**C**) with additional 24 h Amk treatment. White arrows point out bacterial cells with altered morphology. Each panel corresponds to a representative micrograph of two independent experiments.

Scanning electron microscopy (SEM) was the chosen method for analyzing the morphological changes on the *P. aeruginosa* sessile cells exposed to the different antimicrobial treatments (Figure 4). *P. aeruginosa* cells in control macrocolonies exhibited a typical bacillary morphology, closely packed and surrounded by an exopolymeric matrix, which appeared as a roughness cell surface (Figure 4A). When *P. aeruginosa* biofilms were exposed to 100 μg/mL amikacin, a few cells with altered morphology and clear signs of cellular collapse were observed (Figure 4B). A higher incidence of damaged bacteria was noted when the amikacin treatment was followed by aPDT with 600 µM MB (Figure 4C). The treatment that induced the most pronounced and widespread alterations in the majority of the biofilm cells was the sequential exposure to amikacin–aPDT–amikacin (Figure 4D).

### 3.2. Assessment of P. aeruginosa–E. faecalis Dual-Species Biofilm Susceptibility to Antimicrobial Treatments

#### 3.2.1. Effect of Amikacin and Ampicillin on Cell Viability of Established *P. aeruginosa–E. faecalis* Dual-Species Biofilms

As mentioned in the Introduction, our microbiological survey in DFUs evidenced a high frequency of co-isolation of *P. aeruginosa* with *E. faecalis* [5]. For this reason, dual-species biofilms formed by the co-isolated strains Pa036 and Ef 037 were investigated. First, CBM in planktonic mixed cultures to amikacin and ampicillin were determined. Results evidenced that both species showed different susceptibility to these antibiotics, with amikacin being more effective to kill Pa036 than Ef037 whereas the opposite effect was observed with ampicillin (Table 1).

**Figure 5:**
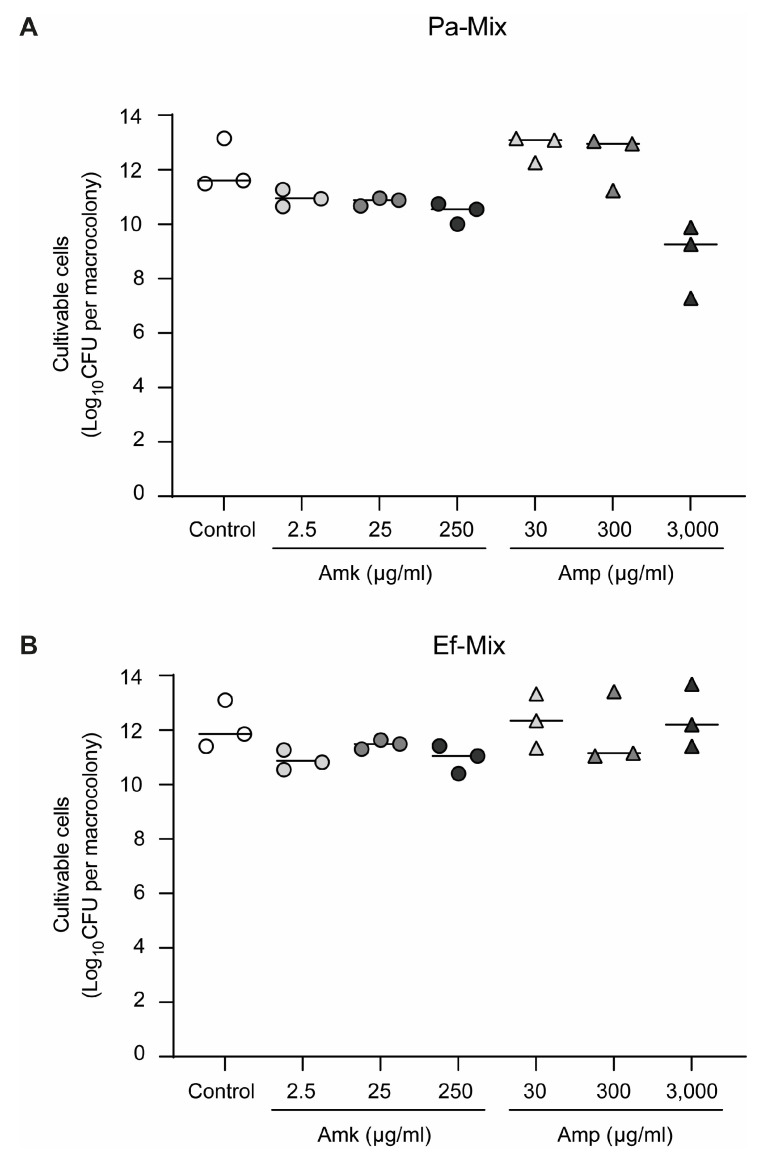
Effect of antibiotic exposure on cell viability of dual-species *P. aeruginosa*–*E. faecalis* macrocolony biofilms. Macrocolony biofilms developed on membranes placed on Lubbock-Glc agar during 24 h, exposed for 24 h to increasing concentrations of amikacin (Amk) or ampicillin (Amp). Control corresponds to biofilms placed on MH agar without antibiotic. Viable cell enumeration of *P. aeruginosa* Pa036 (**A**) and *E. faecalis* Ef037 (**B**) of mixed biofilm-associated bacteria is shown. In each case, N = 3 independent experiments. No significant differences among groups were found after one-way ANOVA analysis followed by Tukey’s post hoc test.

Then, dual-species biofilms developed for 24 h in Lubbock-Glc-agar as explained in Section 3.1.1 were exposed to various amikacin and ampicillin concentrations for 24 h. Results indicated that control macrocolonies were composed of similar numbers of *P. aeruginosa* and *E. faecalis* cells (12.0 ± 1.2 and 12.1 ± 0.9, respectively) (Figure 5). Macrocolonies exposed to increasing concentrations of amikacin exhibited a modest reduction in *P. aeruginosa* viability compared with untreated controls, reaching decreases of approximately 2 Log_10_ CFU/macrocolony at 250 μg/mL amikacin (Figure 5A). In contrast, no reduction in the number of culturable *E. faecalis* cells was detected in mixed-species biofilms exposed to the same amikacin concentrations, nor upon treatment with ampicillin at concentrations of up to 3000 μg/mL (Figure 5B). Notably, Pa036 biofilms showed an average reduction of 3 Log_10_ CFU/macrocolony at the highest ampicillin concentration tested (3000 μg/mL), whereas lower concentrations had no measurable impact on biofilm viability (Figure 5A).

**Figure 6:**
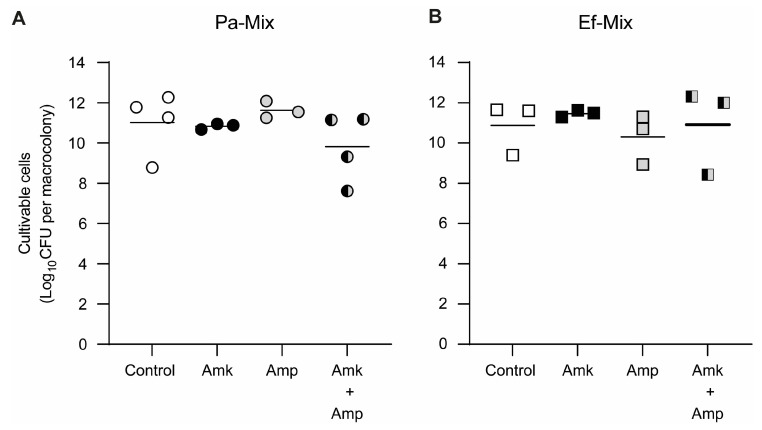
Effect of combined antibiotic exposure on cell viability of dual-species *P. aeruginosa*–*E. faecalis* macrocolony biofilms. Macrocolony biofilms developed on membranes placed on Lubbock-Glc agar during 24 h, exposed to 25 μg/mL amikacin (Amk), 75 μg/mL ampicillin (Amp), or the combination of both antibiotics (Amk + Amp). Control corresponds to biofilms placed on MH agar without antibiotic. Viable cell enumeration of *P. aeruginosa* Pa036 (**A**) and *E. faecalis* Ef037 (**B**) of mixed biofilm-associated bacteria is shown. In each case, N = 3 independent experiments. No significant differences among groups were found after one-way ANOVA analysis followed by Tukey’s post hoc test.

When the combination of amikacin and ampicillin (25 and 75 μg/mL, respectively) was tested on mixed-species biofilms, effects comparable to those observed with each antibiotic alone were detected for both Pa036 (Figure 6A) and Ef037 (Figure 6B). At the concentrations evaluated, no significant changes in cell viability were observed for either Pa036 or Ef037 relative to the untreated control.

#### 3.2.2. Susceptibility of *P. aeruginosa–E. faecalis* Dual-Species Biofilms to MB-aPDT

**Figure 7:**
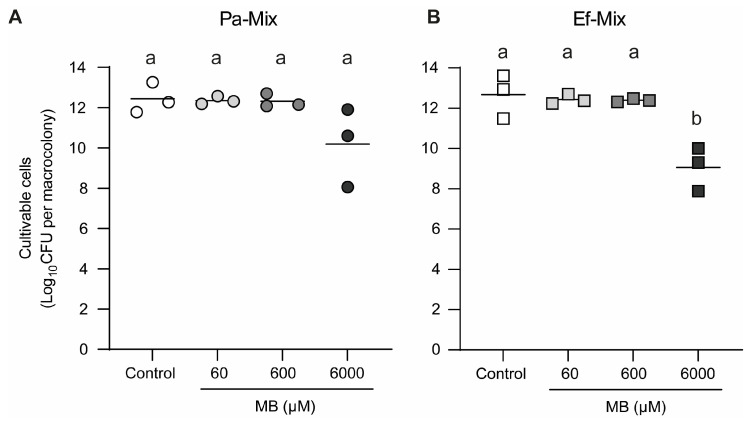
Effect of MB-mediated aPDT on cell viability of dual-species *P. aeruginosa*–*E. faecalis* macrocolony biofilms. Macrocolony biofilms developed on membranes placed on Lubbock-Glc agar during 24 h, exposed or not to aPDT with increasing MB concentrations as explained in legend of Figure 2. Viable cell enumeration of *P. aeruginosa* Pa036 (**A**) and *E. faecalis* Ef037 (**B**) of mixed biofilm-associated bacteria is shown. In each case, N = 3 independent experiments. Different letters indicate *p* < 0.05 assessed by one-way ANOVA followed by Tukey’s post hoc test.

Application of aPDT using MB concentrations of 60 and 600 μM did not result in significant changes in CFU counts of either Pa036 or Ef037 in mixed biofilms compared with untreated control macrocolonies (Figure 7). In contrast, exposure to 6000 μM MB led to a marked reduction in CFU counts for both species. Specifically, Pa036 viability in mixed biofilms decreased by 2.2 Log_10_ CFU (Figure 7A), while Ef037 exhibited a 3.6 Log_10_ CFU reduction in viability relative to the control (Figure 7B).

#### 3.2.3. Sequential Challenge of Mixed Biofilms with Antibiotics and MB-aPDT

**Figure 8:**
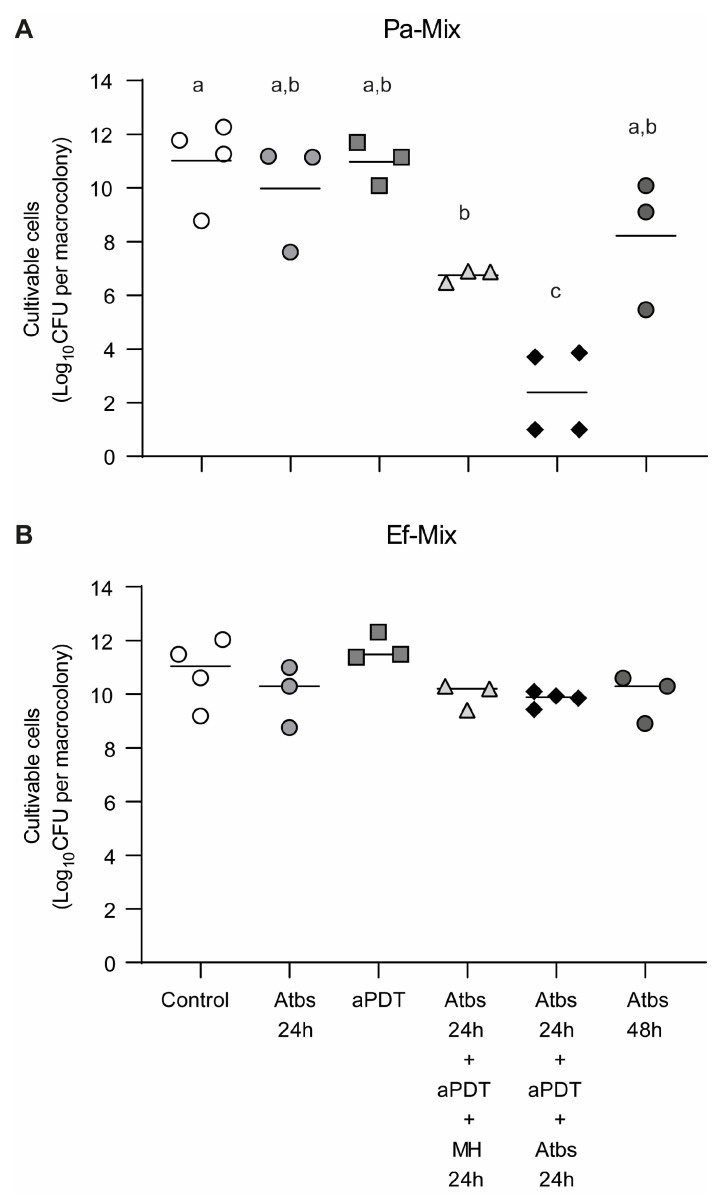
Effect of sequential exposure to antibiotics and MB-mediated aPDT on cell viability of dual-species *P. aeruginosa*–*E. faecalis* macrocolony biofilms. Macrocolony biofilms developed on Lubbock-Glc agar were subjected to individual antimicrobial treatments as described in legends of Figure 6 (25 μg/mL Amk + 75 μg/mL Amp) and Figure 7 (aPDT with MB 600 μM), and sequential applications. Viable cell enumeration of *P. aeruginosa* Pa036 (**A**) and *E. faecalis* Ef037 (**B**) of mixed biofilm-associated bacteria is shown. In each case, N ≥ 3 independent experiments. Different letters indicate *p* < 0.05 assessed by one-way ANOVA followed by Tukey’s post hoc test.

Sequential antibiotic exposure and aPDT assays were performed on mixed Pa036/Ef037 biofilms (Figure 8). The combined effect of the previously tested antibiotics (amikacin, 25 μg/mL, and ampicillin, 75 μg/mL) was evaluated, followed by aPDT using 600 μM MB.

The most pronounced reduction in Pa036 viability was observed following sequential treatment with antibiotics–aPDT–antibiotics, yielding viable counts of 2.4 ± 1.6 Log_10_ CFU in treated biofilms compared with 11.0 ± 1.6 Log_10_ CFU in untreated controls, corresponding to an 8.6 Log_10_ CFU reduction in viability (Figure 8A). When mixed biofilms were exposed to antibiotics followed by aPDT and subsequently incubated in antibiotic-free medium, Pa036 viability decreased to 6.8 ± 0.2 Log_10_ CFU, representing a reduction of approximately 3.2 Log_10_ CFU relative to the untreated control. A comparable decrease (≈3.2 Log_10_ CFU) was observed after 48 h of antibiotic treatment alone.

In contrast, Ef037 viability in mixed biofilms was not significantly affected by any of the treatments tested (Figure 8B).

#### 3.2.4. Scanning Electron Microscopy Analysis of Mixed *P. aeruginosa–E. faecalis* Biofilms Following Combined Antimicrobial Treatment

**Figure 9:**
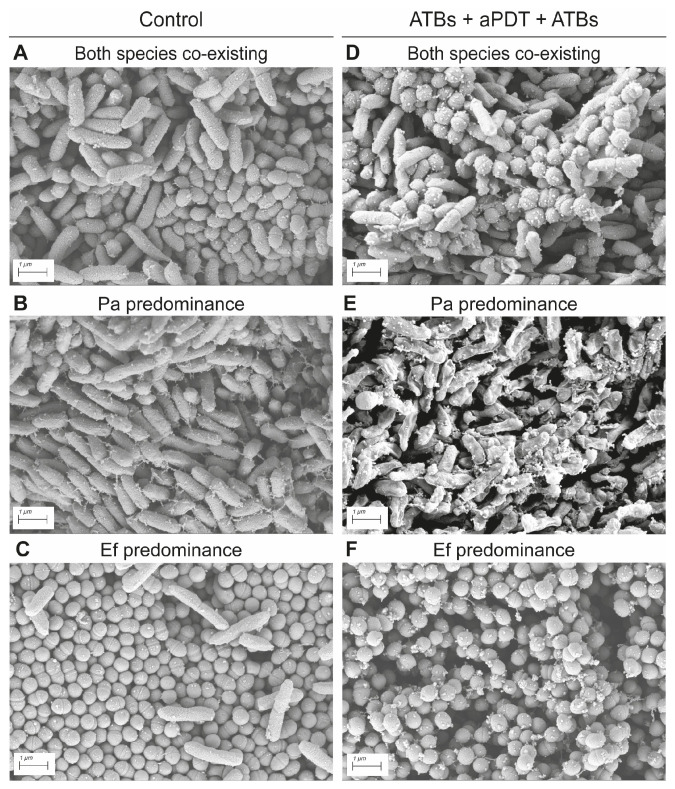
Scanning electron micrograph of dual-species *P. aeruginosa*–*E. faecalis* macrocolony biofilms subjected to different antimicrobial treatments. Micrographs correspond to control (**A**–**C**) and treated (**D**–**F**) biofilms. Sequential Amk–aPDT–Amk treatment was performed as explained in legend of Figure 8. Images were selected to illustrate differential species distribution in mixed biofilms. Each panel corresponds to a representative micrograph of two independent experiments.

SEM analysis of mixed Pa036/Ef037 biofilms revealed the presence of regions where both species were in close contact (Figure 9A), as well as areas dominated by a single species, either *P. aeruginosa* (Figure 9B) or *E. faecalis* (Figure 9C). Sequential exposure of these biofilms to antibiotics (using a combination of amikacin and ampicillin) followed by aPDT and a second exposure of antibiotics produced adverse effects exclusively on Pa036, primarily in regions where this species predominated (Figure 9E). In contrast, Pa036 cells located in close proximity to Ef037 exhibited largely unaltered morphology (Figure 9D). Notably, Ef037 morphology remained unaffected under all observed conditions (Figure 9E–F).

## 4. Discussion

Biofilms represent a bacterial phenotype that is particularly difficult to eradicate because of their high tolerance to both host immune defenses and antimicrobial agents. As a result, biofilms play a key factor in the persistence of DFU infections and help explain the frequent failure to achieve effective wound healing [9]. From a broader perspective, biofilm-associated infections constitute a major global public health concern, as their elevated tolerance to multiple antibiotics markedly compromises the efficacy of conventional antimicrobial therapies. Notably, bacterial growth within biofilms can increase antibiotic tolerance by up to three orders of magnitude compared with planktonic cells [21].

In our in vitro assays, preformed biofilms of *P. aeruginosa*, in both monomicrobial cultures and in mixed communities with *E. faecalis*, exhibited markedly reduced susceptibility to antibiotic treatments that were otherwise effective against their planktonic counterparts. Specifically, exposure to amikacin for 24 h, even at concentrations equal to or exceeding the planktonic minimum bactericidal concentration (MBC) for *P. aeruginosa*, resulted in only a modest reduction in biofilm-associated bacterial viability, not surpassing 2 Log_10_ CFU. Moreover, this limited antibiofilm activity was not dose dependent, as comparable decreases in viability were observed at both 1× and 100× the amikacin MBC.

Given the limited efficacy of antibiotics against biofilm-associated bacteria, antimicrobial photodynamic therapy (aPDT) was evaluated as an alternative strategy for biofilm control. This approach is considered to have a low potential for inducing resistance, likely because the reactive oxygen species (ROS) generated during aPDT simultaneously target multiple cellular structures and metabolic pathways, leading to widespread microbial damage. In contrast, conventional antibiotics typically act on a single or limited number of specific molecular targets, which facilitates the development of resistance mechanisms [22].

Among the available photosensitizing agents, methylene blue (MB) is one of the most widely used due to its low toxicity, making it a safe and efficient option in PDT treatments [14]. MB is activated by absorbing light in the 600–700 nm range. Accordingly, in our study, irradiation was performed using an array of red light-emitting diodes (LEDs) with a maximum emission wavelength (λ_max_) of 639 nm. Biofilm samples were exposed for 2 h at an irradiance of 44 W/m^2^, corresponding to a total light dose of 32 J/cm^2^.

Several studies have demonstrated that antimicrobial photodynamic therapy (aPDT) using methylene blue (MB) effectively reduces the viability of both Gram-negative and Gram-positive bacteria in planktonic cultures, including *P. aeruginosa* [19,23] and *E. faecalis* [24,25]. In contrast, our results indicate that, in biofilm-associated cells, aPDT with MB induced a significant reduction in viability only when a high photosensitizer concentration (6000 μM) was applied. Moreover, this effect was more pronounced in *P. aeruginosa* monomicrobial biofilms than in mixed-species biofilms with *E. faecalis*.

The reduced efficacy observed in mixed biofilms may be attributed, at least in part, to interspecies interactions that alter biofilm architecture. In polymicrobial communities, changes in the composition and organization of the extracellular polymeric matrix can lead to a denser physical barrier, thereby limiting the diffusion of both the photosensitizer and molecular oxygen required for the generation of reactive oxygen species (ROS) [26]. However, because these structural and diffusional changes were not directly assessed in the present study, this interpretation remains indirect and should be validated in future work using approaches that directly probe matrix composition and transport properties.

Decreasing the MB concentration to 600 μM in our experimental conditions led to a loss of the bactericidal effect of aPDT. However, previous studies have reported bactericidal activity against *P. aeruginosa* biofilms using substantially lower MB concentrations (≈250 μM), combined with light doses of 25–30 J/cm^2^ at 660 nm, achieving reductions of approximately 3 Log_10_ CFU [27,28]. Notably, these studies found no further enhancement of antibiofilm activity upon increasing the photosensitizer concentration [28]. Such discrepancies may reflect strain-dependent differences in *P. aeruginosa* biofilm susceptibility to aPDT under comparable irradiation conditions. In this context, the addition of adjuvants such as hydrogen peroxide or ethanol has been proposed as a strategy to enhance aPDT efficacy [27,28].

With respect to polymicrobial biofilms relevant to infected wounds, the effects of MB-mediated aPDT remain insufficiently explored. Available studies have primarily focused on multispecies biofilms associated with oral infections or medical devices, where variable degrees of efficacy have been reported [29,30].

For the final antimicrobial strategy evaluated, sublethal conditions were selected for the individual treatments with antibiotics and aPDT in order to assess the effect of their combined application. The most effective regimen consisted of the sequential administration of antibiotic (amikacin for single-species *P. aeruginosa* biofilms and a mixture of amikacin and ampicillin for mixed biofilms), followed by aPDT, and a subsequent second antibiotic challenge. This protocol was highly effective against *P. aeruginosa* in both monomicrobial and mixed biofilms, resulting in a reduction in cell viability close to eradication in monomicrobial biofilms. In contrast, when the second antibiotic challenge was replaced by incubation in antibiotic-free medium, although a significant loss of *P. aeruginosa* viability was still observed compared with each individual treatment, it was significantly lower than that achieved with the full sequential protocol. This trend was observed in both single-species and mixed biofilms. Overall, these findings suggest that the sequential antibiotic–aPDT treatment enhances antibiofilm activity relative to individual therapies, and that maintaining antibiotic exposure contributes to a greater decrease in bacterial viability. On the other hand, *E. faecalis* within mixed biofilms was not significantly affected by any of the combined treatments tested.

Several studies have demonstrated that the combination of aPDT and antibiotics represents a promising strategy for the treatment of biofilm-associated bacterial infections, as it can target multiple cellular and structural components simultaneously [31,32,33]. This approach has been proposed to weaken the extracellular polymeric matrix and enhance antibiotic penetration and activity within the biofilm [31]. The generation of reactive oxygen species during PDT may contribute both to direct bacterial killing and to biofilm disruption, thereby exposing previously protected cells. As a result, antibiotics—even when administered at lower concentrations—can exert a stronger effect on the surviving bacterial population, resulting in synergistic antimicrobial activity that exceeds the efficacy of either treatment applied alone [34,35].

It is noteworthy that the antimicrobial efficacy of the simultaneous application of aPDT and antibiotics has been widely reported for both planktonic cultures and biofilms [35,36,37]. In contrast, studies addressing the antimicrobial effectiveness of sequential treatments, particularly aPDT followed by antibiotics, remain limited. In this context, the work by Zangirolami et al. (2024) is of particular interest, as it describes the sequential application of gentamicin, an aminoglycoside antibiotic, for 6 h followed by aPDT in planktonic cultures of *P. aeruginosa*, and highlights that this regimen results in a synergistic antimicrobial effect compared with the individual treatments [38]. Consistently, our results show an enhanced antibiofilm effect against *P. aeruginosa* when another aminoglycoside, amikacin, is applied sequentially with aPDT.

Unexpectedly, SEM analysis of mixed biofilms revealed marked heterogeneity in the spatial distribution of the two bacterial species. In regions where *P. aeruginosa* was in close proximity to *E. faecalis*, the combined treatment did not induce the pronounced morphological alterations observed in areas enriched exclusively with *P. aeruginosa*. These observations suggest a potential protective effect exerted by *E. faecalis* on *P. aeruginosa*.

Further research will be relevant to clarify the mechanisms underlying the synergy between aPDT and antibiotics. In particular, transcriptomic and proteomic analyses under the different experimental conditions would help to elucidate ROS-mediated damage to proteins and lipids, as well as the regulation of genes involved in stress responses, cell wall integrity, metabolic activity, and biofilm maintenance. In addition, such approaches could clarify how antibiotics influence bacterial physiology in this context, including effects on membrane permeability, metabolic pathways, and tolerance mechanisms within biofilms [39].

It is worth noting that aPDT employing red light (619–780 nm) offers an additional advantage in the treatment of skin lesions, as it can modulate several biological processes through photobiomodulation. This phenomenon arises from the absorption of light by intracellular chromophores and, through photochemical and photothermal mechanisms, enhances mitochondrial activity, increases cellular energy production, improves tissue perfusion, attenuates inflammatory responses, and stimulates collagen synthesis and cell regeneration in the affected area, thereby promoting tissue healing [40].

Finally, the clinical relevance of this strategy lies in its potential to overcome the intrinsic tolerance of biofilms to conventional antibiotics, which remains one of the major challenges in current antimicrobial therapy. Moreover, the possibility of reducing antibiotic doses without compromising therapeutic efficacy is particularly relevant in light of the global rise in antimicrobial resistance. Notably, the outcome of antibiotic–PDT combinations appears to be strongly dependent on the antibiotic class, as certain agents exhibit synergistic interactions with PDT, whereas others may display antagonistic effects [34]. Given that these observations are derived from in vitro experimental systems, their translational relevance remains to be established in more complex and clinically representative models.

Taken together, our findings support the combination of aPDT and antibiotics as a promising and innovative strategy for the management of persistent infections caused by multidrug-resistant pathogens, such as *P. aeruginosa*.

## 5. Conclusions

In this study, we show that *Pseudomonas aeruginosa* biofilms, formed either as monomicrobial communities or mixed-species with *Enterococcus faecalis*, under conditions mimicking diabetic foot ulcer infections, display substantial tolerance to conventional antibiotic therapy and limited susceptibility to methylene blue-mediated antimicrobial photodynamic therapy (aPDT) when these approaches are applied individually. Notably, the sequential combination of antibiotics and aPDT was associated with an enhanced antibiofilm effect against *P. aeruginosa*, leading to near-complete eradication in monomicrobial biofilms and marked reductions in mixed-species biofilms. In contrast, *E. faecalis* remained unaffected, underscoring species-specific responses and the role of polymicrobial interactions in shaping treatment outcomes. Overall, these results suggest that combined antibiotic–aPDT strategies may help improve antimicrobial efficacy against biofilm-associated infections relevant to diabetic foot infections, while also highlighting the importance of further studies to clarify interspecies protective mechanisms and to optimize treatment protocols for polymicrobial biofilms.

## Figures and Tables

**Table 1:** Minimal bactericidal concentrations (MBC) of amikacin and ampicillin on mixed Pa036—Ef037 planktonic cultures.

	MBC (μg/mL)	
	Amk	Amp
Pa036-Mix	1.25	>240
Ef037-Mix	100	30

Values are the mean of three independent experiments.

## Data Availability

The original contributions presented in this study are included in the article. Further inquiries can be directed to the corresponding author.
